# Feed gap analysis of dual-purpose chicken production in Tanzania: feed quantity and quality limited production

**DOI:** 10.1016/j.psj.2023.102574

**Published:** 2023-02-09

**Authors:** Wilson C. Wilson, Maja Slingerland, Frederick P. Baijukya, Ken E. Giller, Simon Oosting

**Affiliations:** ⁎ Plant Production Systems Group, Wageningen University, 6700 AK Wageningen, the Netherlands; † Animal Production Systems Group, Wageningen University, 6700 AH Wageningen, the Netherlands; ‡ International Institute of Tropical Agriculture (IITA), Dar es Salaam, Tanzania; § Tanzania Livestock Research Institute (TALIRI), Uyole Centre, Mbeya, Tanzania

**Keywords:** productivity, feed ingredient, feed quantity, aflatoxin

## Abstract

The demand for chicken meat and eggs exceeds what can be produced in Tanzania, largely due to low productivity of the sector. Feed quantity and quality are the major factors determining the potential production and productivity of chickens. The present study explored the yield gap in chicken production in Tanzania and analyses the potential of increased chicken production as a result of closing the feed gaps. The study focused on feed aspects limiting dual-purpose chicken production in semi-intensive and intensive systems. A total of 101 farmers were interviewed using a semistructured questionnaire and the amount of feed provided to chickens per day was quantified. Feed was sampled for laboratory analysis and physical assessments were made of weights of chicken bodies and eggs. The results were compared with the recommendations for improved dual-purpose crossbred chickens, exotic layers, and broilers. The results show that the feeds were offered in insufficient quantity compared with the recommendations for laying hens (125 g/chicken unit/d). Indigenous chickens were fed 111 and 67 while the improved crossbred chickens were fed 118 and 119 g/chicken unit/d under semi-intensive and intensive systems, respectively. Most feeds fed to dual-purpose chickens were of low nutritional quality, particularly lacking in crude protein and essential amino acids in both rearing systems and breeds. Maize bran, sunflower seedcake, and fishmeal were the main sources of energy and protein in the study area. The study findings show that the important feed ingredients: protein sources, essential amino acids, and premixes were expensive, and were not included in formulating compound feeds by most chicken farmers. Of all 101 respondents interviewed, only one was aware of aflatoxin contamination and its effects on animal and human health. All feed samples contained a detectable concentration of aflatoxins and 16% of them exceeded the allowed toxicity thresholds (>20 µg/kg). We highlight the need for a stronger focus on feeding strategies and ensuring the availability of suitable and safe feed formulations.

## INTRODUCTION

In Tanzania, human nutrition can be improved through the consumption of affordable locally produced poultry products. Chickens are the predominant poultry species in Tanzania raised by 86% of livestock-keeping households, supporting livelihoods, and providing nutrient-dense animal-sourced protein and income along the value chain (MLDF, 2015). Most consumers in Tanzania prefer indigenous chicken meat and eggs to products from exotic broilers and layers (Naggujja et al., 2020) due to the perception that they taste good and are nutritious and that they come from chickens which are raised organically (Sanka and Mbaga, 2014; Muhikambele, 2019). Producers also prefer indigenous breeds because of their resilient nature to diseases and to harsh conditions which result in less need for veterinary drugs (Muhikambele, 2019; Kapella et al., 2022). The demand for chicken meat and eggs is already high in Tanzania, and it is projected to increase. It is challenging to meet the increasing demand through domestic production (MLDF, 2015; Naggujja et al., 2020) due to the low production capacity of indigenous breeds and limited access to quality feeds.

Over the past decade, the chicken industry in Tanzania intensified, involving the transition from keeping a small number of free-range indigenous chickens to keeping improved dual-purpose crossbred and exotic layers and broilers in semi-intensive and intensive systems (Ringo and Lekule, 2020; Wilson et al., 2022). Hence, chicken production systems in Tanzania are categorized into 3 rearing systems, that is, free-range, semi-intensive, and intensive. These different rearing systems are primarily defined by the housing and feeding systems. Free-range is scavenging outdoors, the semi-intensive system is partly indoors and fed with home-made feed, while in the intensive system the chickens are permanently indoors and fed with commercial or home-made feed or both (Wilson et al., 2022).

Feed quality and quantity are major aspects determining the production and productivity of chickens. The theoretical concepts of production ecology have been applied in assessing the potential, limited, and actual crop and animal production (Van de Ven et al., 2003; van der Linden et al., 2015). When applied to animal production, potential production is defined by the growth-defining factors, that is, genotype and climate, while limited production is defined by the growth-limiting factors, that is, feed quantity and nutritional quality (van der Linden et al., 2015). The actual production is defined by growth-reducing factors, that is, diseases and parasites, and feed contamination (e.g., by a mycotoxin such as aflatoxin). Using this approach, the yield gap, which is the difference between the actual and limited yield or potential yield can be estimated. Moreover, in practice, research on yield gap analysis shows that is not feasible in practice or cost-effective to close the yield gap fully (van Ittersum et al., 2013). The exploitable yield gap in crop and livestock production is therefore between 75 and 85% of potential production (van Ittersum et al., 2013; van der Linden et al., 2015).

The Tanzania Livestock Master Plan (**TLMP** 2017–2022) highlights that limited access to quality feeds and feed ingredients is among the major constraints to the development of the poultry sector in the country (Nandonde et al., 2017; MLDF, 2017) and hence is a major contributor to the yield gap. The TLMP further highlights the potential of maize and soybean as local sources of energy and protein, respectively, in chicken feed, providing an opportunity for growth of the chicken industry in the country. Despite the low maize and soybean yield in Tanzania (as for most sub-Saharan African countries), there is enormous potential to reduce the yield gap through improved agronomic practices (Van Loon et al., 2018; Giller et al., 2021). Moreover, the country potentially has about 44 million hectares of land suitable for crop production, of which only 24% is being used for farming at present. So, extra land can be made available for food and feed production while considering sustainable land use strategies (Msuya, 2013; Msofe et al., 2019).

Chicken feed accounts for about 70% to the production costs in Tanzania (Mutayoba et al., 2011). Commercial feeds are expensive and mostly not available in quantities small enough to be affordable to smallholder chicken farmers (Wilson et al., 2021). Moreover, most farmers rely on local feeds and feed ingredients from local stores and markets. Often, the quality of these locally available feeds is questionable, and the prices, particularly of the protein sources used (e.g., fish meal), are high (Nandonde et al., 2017; Andrew et al., 2019). Quality control of chicken feed and feed ingredients along the supply chain is limited, while there is no labeling of feed packages by processors showing the nutrient contents of feeds (Geerts, 2014). Moreover, there is little compliance with recommended feed standards due to lack of awareness, and weak extension services (Longo Joseph, 2019; Matrona et al., 2022). There are recent reports of high mycotoxin contamination in feeds and feed ingredients, which arises during crop production and storage (Suleiman et al., 2017; Agape et al., 2021; Temba et al., 2021), posing risks to chicken and human health (Shephard, 2008). Therefore, feed availability and quality are major contributors to the feed gap in poultry production in Tanzania.

In the present paper, we focus on this specific component of yield-gap analysis, namely an analysis of the so-called “feed gap.” A feed gap analysis is the comparison of the actual feed quantity and quality supplied to chickens with the recommended standards for improved dual-purpose crossbred chickens, and exotic layers and broilers. We explore the current feed gap and how this feed gap can be closed. The study focuses particularly on farms keeping indigenous and improved dual-purpose crossbred chickens raised for both meat and eggs under semi-intensive and intensive systems in the Southern Highlands of Tanzania.

## MATERIALS AND METHODS

### The Study Area

The study was conducted in Iringa municipality in the Iringa region, Southern Highlands of Tanzania. The municipality covers urban and periurban locations with limited land for crop production and is characterized by semi-intensive and intensive chicken production systems (Wilson et al., 2022). Furthermore, the municipality has potential for commercialization of the chicken industry due to presence of hatcheries, grain millers and feed processors established over the past decade.

### System Boundaries

**Figure 1:**
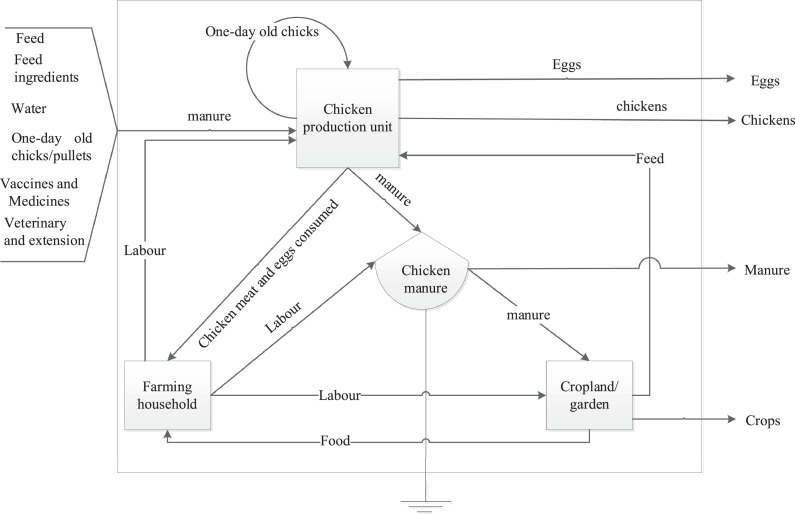
System description for farms raising dual-purpose chickens in urban and periurban locations, Iringa region, Tanzania.

Yield gap analysis in the present study was done at the farm level. A farm is defined as an entity comprised of a farming household with a chicken production unit and cropland or garden. The studied farms in urban and periurban areas relied on external inputs, that is, 1-day-old chicks, feed or feed ingredients, water, medicines and vaccines, and extension services (Figure 1) (Wilson et al., 2022). Some farms produced chicks themselves and had access to chicken feed from their own cropland or garden. The farming household members provided labor to manage both the chicken production unit and cropland. Chicken meat and eggs were both consumed within the farm and sold. Chicken manure in small-scale farms is mainly used within the farms while some mid-large-scale farms sell manure to crop farmers. On the other hand, excess manure from landless farms is dumped in the garbage area.

### Sampling Framework and Data Sources

#### Primary Data

The actual production and feed quantity and quality (status quo) were primarily assessed by a cross-sectional survey, physical measurements, quantification of feed offered to chickens during a farm visit and feed quality assessment by laboratory analysis.

A cross-sectional survey was executed in the Iringa Municipality from November to December 2020 whereby 101 farmers keeping dual-purpose chickens were interviewed using a semistructured questionnaire programmed using Open Data Kit (**ODK**) software (https://opendatakit.org/). A stratified sampling of farms was conducted in 14 Wards in the municipality. The wards were purposively selected based on having high number of dual-purpose chickens according to the extension office statistics. To better understand the diversity of chicken production and feeding practices, the wards within the municipality were subdivided based on the location of the farming household along the urban-periurban gradient, that is, the urban sublocation (Group I: <5 km from the town center) constituting of 8 Wards, and the periurban sublocation (Group II: ≥5 km from the town center), constituting of 6 Wards. A random sample of farms keeping dual-purpose chickens was selected from the 2 sublocations based on the list provided by the extension office, resulting in 45 and 56 farms in urban and periurban sublocations, respectively. The interviews were conducted by trained enumerators. The interviewees were adult respondents directly involved in chicken management. Chicken production systems were grouped into 2 predefined rearing systems, that is, semi-intensive and intensive systems, primarily based on housing, and feeding system (Wilson et al., 2022). The majority of farmers raised chickens in semi-intensive systems (81%) and a minority were practicing intensive systems (19%). Of all farms, 64% raised indigenous and 36% improved crossbred chickens (Table 1), while 5% raised multiple breeds of chicken in 1 or more rearing systems. The questionnaire included questions about the management of chickens, type of feed and feed ingredients used in formulating chicken feed, sources of feed, feed availability, prices of feed and feed ingredients, and the quantities and prices of the products produced and marketed (i.e., meat, eggs, and manure).

**Table 1:** Distribution of interviewed poultry farmers and feed sample collection in Iringa municipality.

	Rearing system		
	Semi-intensive	Intensive	Total number of farms
Indigenous breed	54	11	65
Improved crossbred	28	8	36
Total	82	19	101

During the farm visits, physical measurements were taken for assessment of the performance of chickens. Measurements taken include live body weight and egg weight, recorded using a digital weighing scale in grams; and shank length and circumference measured using a tailoring and a digital vernier caliper, respectively. Shank length and thickness or circumference are important parameters in measuring the skeletal development of chickens and they also have a direct relation with growth and body weight (Yakubu et al., 2009; Mutayoba et al., 2012). Physical measurements were done in all 101 farms interviewed whereby 4 to 5 chickens and eggs were randomly selected for physical measurements in each farm, depending on the flock size and number of eggs at the moment of farm visit. In total, 401 hens and 462 eggs were selected for physical measurements.

Feed quantity and quality were also assessed during the farm visits. The quantity of feed fed to chickens per day, feed formulation practices and storage, and seasonal availability of different feed ingredients used in feed formulation were recorded. The feed offered was estimated based on a measurement of the total amount fed to the whole flock on the day of the farm visit. The production parameters assessed included the number of laying hens per farm, the number of eggs per hen/week and the body weight of chickens.

Feed quality characteristics, that is, nutritional quality and aflatoxin content were assessed in feed samples (one sample from each farm, sampled from the whole mixed ration) which were collected from semi-intensive (78 farms) and intensive systems (19 farms) and analyzed for dry matter (**DM**), crude protein (**CP**), crude fat, crude fiber (**CF**), ash, metabolizable energy (**ME**), and essential amino acids (lysine, methionine, and tryptophan). Of the 101 interviewed farms, 4 farms had already fed the last feed portion to chickens on the day of the farm visit and were not sampled for laboratory analysis. The nutritional quality of feed was done using Near-Infrared Reflectance Spectroscopy (**NISRS**) at the Tanzania Veterinary Laboratory Agency (**TVLA**) animal feeds laboratory, by exposing the feed sample to an electromagnetic scan over a spectral wavelength range of 1,100 to 2,500 nm (Corson et al., 1999). Thirty-two feed samples constituting 23 samples from semi-intensive and 9 samples from intensive farms were analyzed for aflatoxin contamination using an AccuScan Gold III reader, a single-step lateral flow immunochromatographic assay at the International Institute of Tropical Agriculture (**IITA**) pathology laboratory in Dar es Salaam, Tanzania.

#### Secondary Data

Secondary data were gathered about feed quantity, quality, and chicken production. The quality and quantity of the feed supplied to indigenous and improved dual-purpose crossbred chickens were compared to the recommendations in the Hendrix Genetics guidelines (Hendrix Genetics, 2022). The recommendations for layers and broilers differed slightly between sources. We therefore averaged the recommendation from the East Africa Community Standards (TBS, 2020), Feed Calculator App (FeedCalculator, 2022), and Lohman breeder (for layers’ feed) (Thiele and Pottgüter, 2008) and PoultryHub recommendations (for broiler finishers’ feed) (PoultryHub, 2022), respectively (Table 2). The number of chickens were expressed as Chicken Units (**CU**), defined as the equivalent of a mature chicken of 1,800 g.

**Table 2:** Nutritive value of chicken feed based on the East African Standards, Feed Calculator App, Hendrix Genetics guidelines (for Sasso breeder chickens), and Lohmann layer standards.

	Moisture (%)	Crude fat (%)	Crude protein (%)	Crude fiber (%)	Lysine (%)	Methionine/cystine (%)	Tryptophan (%)	ME (kcal/kg)	Aflatoxin (µg/kg)
(a) East Africa standards guidelines^1^									
1. Broiler finisher feed	12	10.9	18.0	7.5	1.0	0.8	1.25*	3000	<20
2. Layer feed	12	7.9	16.0	7.5	0.7	0.6	1.25*	2650	<20
(b) Feed calculator app guidelines									
1. Standard broiler finisher		9.5	19.0	6.6	0.9	0.7		2750	<20
2. Standard layer feed		7.1	16.5	6.5	0.6	0.5		2600	<20
(c) Breeding company's guidelines									
1. Sasso breeder^2^#		>3.5	17	<6	0.7	0.6	0.16	2750	<20
2. Poultry hub^3^			20		1.1	0.8	0.2	3225	
3. Lohmann layer^4^			17.5		0.9	0.7	0.18	2800	<20
Average broiler finisher ((*a*_1_ + *b*_1_ + *c*_*2*_)/3)	12	10.2	19	7.1	1.0	0.8	0.2	2992	<20
Average layer feed ((*a*_2_ + *b*_2_ + *c*_*3*_)/3)	12	7.5	17	7.0	0.7	0.6	0.18	2683	<20

1 Source: Tanzania Bureau of Standards (TBS, 2020). * Extreme high compared to the recommended threshold from breeding companies (excluded in calculating the average recommended value for tryptophan).

2 25th week age (peak production), egg weight 52.3 g, BW = 1,820 g, weekly lay 95%, feeding g/♀/day = 127 g. Source: Hendrix Genetics breeding manual for SASSO chickens (SA51A)^1^. Source: Hendrix Genetics (Hendrix Genetics, 2022)—https://africa.sasso-poultry.com/documents/853/SASSO_Traditional_Poultry_Breeders_SA51A.pdf.

3 Broiler finisher (8 wk of age), BW = 3,700 g (hens), cumulative feed intake = 8,200 g and 100 g/d. Source: (PoultryHub, 2022)—https://www.poultryhub.org/all-about-poultry/nutrition/nutrition-requirements-of-meat-chickens-broilers.

4 21 to 22 wk (layers in phase 1 of laying), egg weight 53 to 62.7 g, BW = 1,730 g, weekly lay 92 to 94%, feeding g/♀/day = 125 g). Source: Lohmann layer (Thiele and Pottgüter, 2008). Particular environments, sanitary conditions, geographic location, or equipment might require adaptations that have not been taken into consideration in these general recommendations.

# Matured improved crossbred Sasso chicken of 1,800 g, at peak egg production (25–26 wk) and 95% weekly lay (Hendrix Genetics, 2022).

### Data Analysis

R package 4.2.1 was used to analyze the effects of the rearing system and breed on production parameters, that is, maturity age, body weight, weekly lay %, egg weight, and physical parameters of shank length and circumference (Table 3), and the differences in feed quantity (per CU) and quality (Table 4). Weekly lay % as an indicator for laying hen productivity was computed by dividing the number of eggs produced in the farm per week divided by the number of laying hens in the flock at that farm divided by 7 (Ibrahim et al., 2019). The generalized linear model (**GLM**) was used to test the effects of the rearing system (semi-intensive and intensive), breed (indigenous and improved cross bred), location type (periurban and urban) and interactions at a 5% level of significance. During data analysis, no significant differences were found between periurban and urban sublocations, and therefore the sublocation effect was not included in the results presented in the current article. The following statistical model was used to analyze the effects of the rearing system and breed of chicken on the performance of chickens (physical parameters):

$$Y_{ijk}=\mu +R_{i}+B_{j}+L_{k}+R_{i}\times B_{j}+R_{i}\times L_{k}+B_{j}\times L_{k}+R_{i}\times B_{j}\times L_{k}+e_{ijk}$$

where *Y_ijk_* is an observation for a given variable; *μ* is an overall mean; *R_i_* is the effects of the *i*th rearing system (1 = semi-intensive, 2 = intensive); *B_j_* is the effect of *j*th breed (1 = indigenous, 2 = improved cross bred); *L_k_* is the effect of *k*th location (1 = urban, 2 = periurban); *R_i_ × B_j_ × L_k_* is the interaction between the rearing system (*i*), breed (*j*), and sublocation (*k*); and *e_ijk_* is the residual error term.

**Table 3:** Production parameters used in assessing the production of the dual-purpose chickens in the Iringa region (mean and standard deviation).

Rearing system	Semi-intensive (*n* = 82)		Intensive (*n* = 19)		*P* values^2^		
Breed	Indigenous (*n* = 54)	Crossbred (*n* = 28)	Indigenous (*n* = 11)	Crossbred (*n* = 8)	System	Breed	System × breed
Flock size (number of chickens)	98.6 ± 117	100 ± 71.7	117 ± 165	92.5 ± 61.2	0.86	0.69	0.66
Number of laying hens	11.2 ± 11.8	17.3 ± 26.1	6.6 ± 5.8	53.3 ± 26.5	**0.00**	**0.00**	**0.00**
Perceived average maturity age of hens (weeks)^1^	23.7 ± 0.4	21.8 ± 0.5	23.6 ± 0.9	21.3 ± 0.9	0.47	**0.00**	0.25
Body weight (g)	1702 ± 385	2479 ± 517	1732 ± 308	2924 ± 819	**0.05**	**0.00**	0.09
Egg weight (g)	43.0 ± 5.5	50.0 ± 7.1	42.7 ± 8.2	55.00 ± 12.5	0.18	**0.00**	0.14
Egg production /flock/week	47.7 ± 48.1	82.1 ± 110	35.8 ± 36.1	208 ± 130	**0.00**	**0.00**	**0.00**
Weekly lay (%)/flock/week	8.0 ± 6.3	13.8 ± 14.0	6.4 ± 3.8	35.7 ± 17.1	**0.00**	**0.00**	**0.00**
Weekly lay (%)/laying hens in the flock/week	59.9 ± 32.1	61.3 ± 29.3	59 ± 23.9	87.8 ± 10.9	0.10	**0.05**	0.08
Shank length (cm)	6.1 ± 0.1	6.3 ± 0.1	6.2 ± 0.1	6.3 ± 0.1	0.29	0.06	0.19
Shank thickness (mm)	13.2 ± 5.9	13.4 ± 8.2	11.8 ± 13.7	13.6 ± 15.4	0.52	0.38	0.72

1 Perceived maturity age of hens (age at first laying) based on the interviews (farmer's memory).

2 *P*-values in boldface indicate a statistically significant difference.

**Table 4:** Mean and standard deviation values and *P* values for nutritional quantity and quality of chicken feed in the study area.

Rearing system	Semi-intensive		Intensive system		*P* values		
Breed	Indigenous (*n* = 54)	Crossbred (*n* = 28)	Indigenous (*n* = 11)	Crossbred (*n* = 8)	System	Breed	System × breed
Feed quantity (g/chicken unit)	111 ± 59	118 ± 60	67 ± 32	119 ± 52	0.14	0.1	0.13
Metabolizable energy (kcal/kg feed)	2735 ± 147	2674 ± 184	2723 ± 162	2671 ± 90	0.86	0.17	0.91
Crude protein (%)	11.9 ± 1.17	12.6 ± 1.13	12.5 ± 1.53	12.7 ± 1.06	0.39	0.19	0.45
Crude fat (%)	8.1 ± 1.31	7.8 ± 0.88	8.4 ± 1.03	8.0 ± 0.81	0.38	0.28	0.84
Crude fiber (%)	5.7 ± 1.38	5.8 ± 1.05	5.8 ± 1.02	5.3 ± 0.66	0.57	0.53	0.27
Lysine (%)	0.69 ± 0.07	0.72 ± 0.06	0.71 ± 0.09	0.72 ± 0.06	0.40	0.18	0.47
Methionine/cystine (%)	0.51 ± 0.05	0.54 ± 0.05	0.53 ± 0.06	0.54 ± 0.05	0.43	0.17	0.50
Tryptophan (%)	0.14 ± 0.01	0.15 ± 0.01	0.15 ± 0.02	0.15 ± 0.01	0.61	0.28	0.43
Moisture content (%)	10.9 ± 1.21	10.7 ± 1.05	10.4 ± 1.43	10.3 ± 0.61	0.12	0.63	0.77
Aflatoxins (µg/kg)^1^	13.1 ± 7.73	8.4 ± 2.87	14.4 ± 11.28	14.6 ± 6.84	0.32	0.54	0.52

1 the sample size for aflatoxin was 32 farms (23 semi-intensive and 9 intensive systems).

In the next step, feed quantity and quality per farm were plotted against the recommended values using the *ggplot2* package in R. The potential weekly lay % was assumed to be 95% weekly lay at peak production (25–26 wk) for the improved crossbred Sasso chickens (Hendrix Genetics, 2022), illustrating the flock size with the actual and potential egg production per flock per week. In practice, a realistic potential weekly lay % is probably between 75 and 85% of the potential production. Therefore, in the current study, the realistic potential weekly lay % (as an indicator of egg productivity) was assumed to be between 80% (van der Linden et al., 2015).

Second, the actual quantity of the feed (per CU) and nutritional quality were compared with the recommendations for the improved dual-purpose crossbred, exotic layers and broilers. Furthermore, the average levels of aflatoxins and moisture content of the feeds were calculated per system and breed (Table 4), and compared with the recommended limits (Table 2).

## RESULTS

### General Characteristics of the Chicken Farming Household

Overall, 61% of the interviewed respondents were women and 39% were men. The majority of the respondents had attained secondary education (62%), while others attended tertiary education, that is, college or university (24%) and primary school education (13%). The most important sources of household income were poultry business (65%), off-farm informal businesses (12%), crop farming (5%), off-farm formal business (4%), and income from salaries (2%). Of all 101 farmers interviewed, 11 were involved in farmer groups, mainly for joint marketing, for accessing information and training about chicken management, and for accessing loans.

### Flock Size, Reproduction, and Production Characteristics

**Figure 2:**
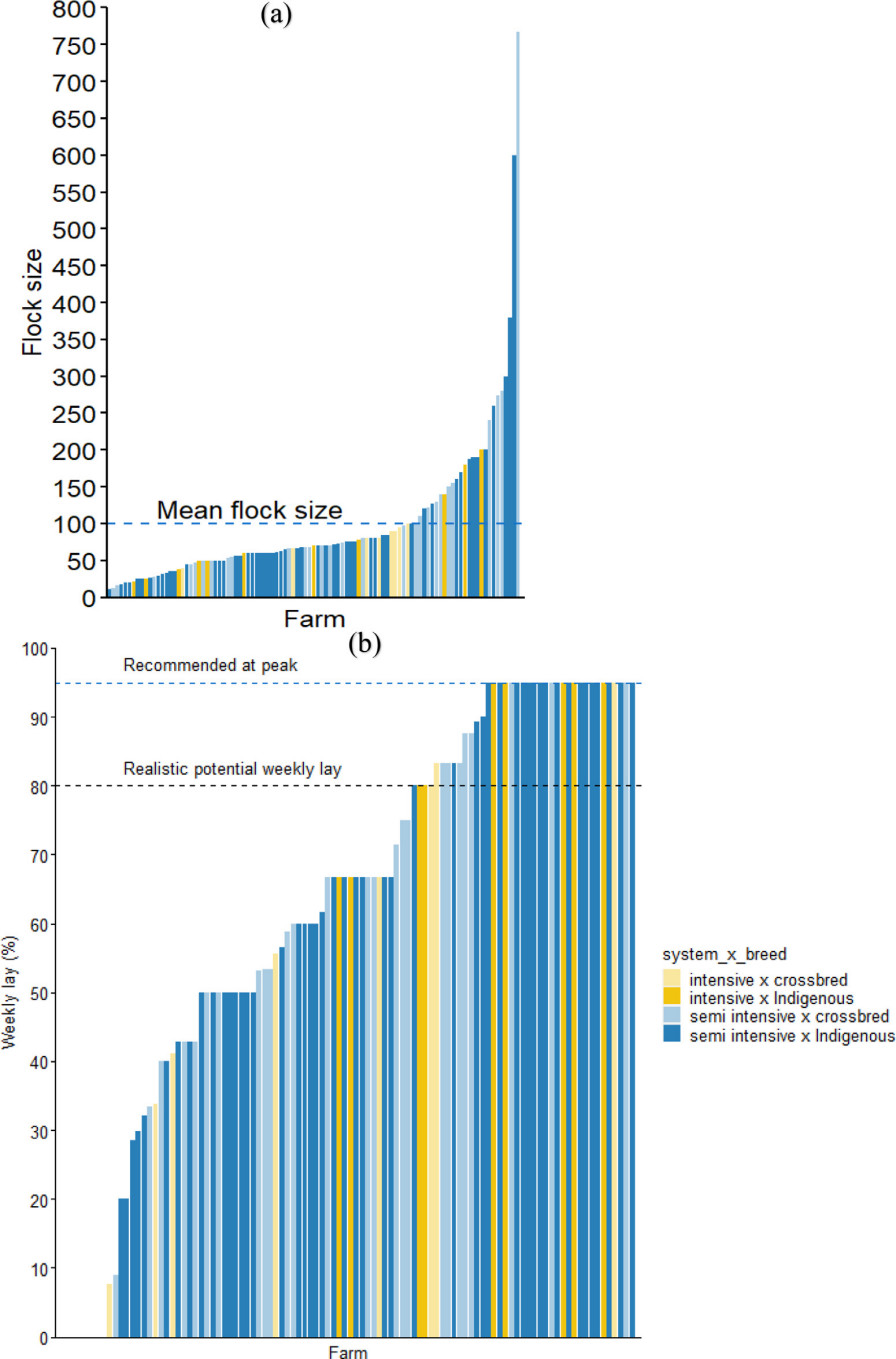
Flock size distribution (a) and weekly lay and weekly lay per laying hens (b) at poultry farms. The recommended percentage at peak (95%) was based on the recommendations for improved crossbred chickens at 25 to 26 wk (Hendrix Genetics, 2022) while the realistic weekly lay percentage is taken to be 80% of potential production (van der Linden et al., 2015).

The overall flock size was 100 chickens per household on average without significant differences between breed and rearing system (Figure 2a and Table 3). The total number of laying hens in the flock was influenced by the rearing system and breed of chickens (*P* < 0.05), where we found that the households that keep improved breeds under the intensive system had more laying hens than households keeping indigenous breeds (*P* < 0.05). Of the 101 interviewed farms, 64% raised indigenous breeds while 36% were keeping the improved crossbred chickens (Table 1). We found that the improved crossbred chickens attained maturity (assessed as the perceived age at first lay) earlier than the indigenous chickens (*P* < 0.05), irrespective of the raising system (Table 3).

Of 401 chickens and 462 eggs sampled for physical measurements, 52 and 21% met the recommended weight for matured crossbred chicken (≥1,800 g) and egg (≥52 g), respectively (Table 2). The body weight of chickens and egg weight were higher (*P* < 0.05) for the improved crossbred than for the indigenous chickens. The number of eggs and weekly percentage lay were influenced by the rearing system and breed of chickens (*P* < 0.05), where we found the largest flocks in households that keep the improved crossbred chickens under the intensive system (Table 3). Sixty percent of the interviewed farming households that keep crossbred chickens reported low weekly lay (Figure 2b), under the intensive and semi-intensive systems. Shank length and circumference were not significant different between production systems and breeds (Table 3).

### Chicken Feed Ingredients and Feeding

**Figure 3:**
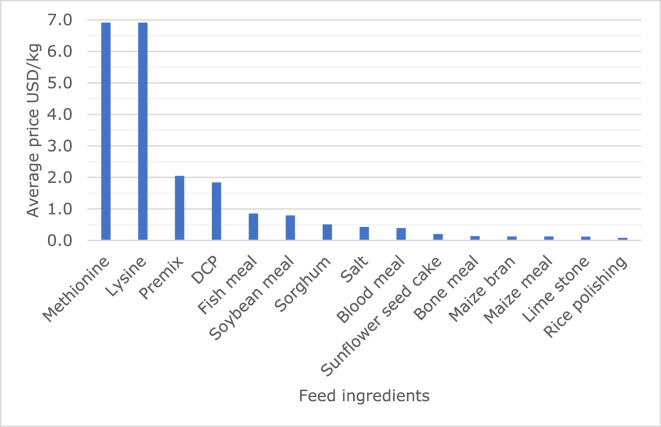
Prices of feed ingredients used for chicken feeding. Abbreviation: DCP, di-calcium phosphate.

The most common feed ingredients used in formulating compound feeds among farms included maize bran and sunflower seedcake, reported by 95 and 93% of the interviewed farmers, respectively. Other feed ingredients reported by farmers (percentage of farmers using the ingredient) included fish meal (68%), limestone (66%), bone meal (60%), di-calcium phosphate (54%), premixes (46%), salt (42%), sorghum (29%), soybean meal (16%), maize meal (11%), rice polishings (10%), blood meal (10%), lysine (2%), and methionine (2%). The use of feed ingredients was determined by price and availability and farmers indicated that the most expensive feed ingredients were less frequently used in formulating feeds. The essential amino acids (methionine, lysine) were mentioned as the most expensive feed ingredients followed by premixes and di-calcium phosphate, but farmers appreciated that they were sold in small quantities (≤0.5 kg). Protein sources, including fish meal and soybean meal, were also expensive: both were sold between 0.7 and 0.9 USD per kg (Figure 3).

### Feed Quantity

**Figure 4:**
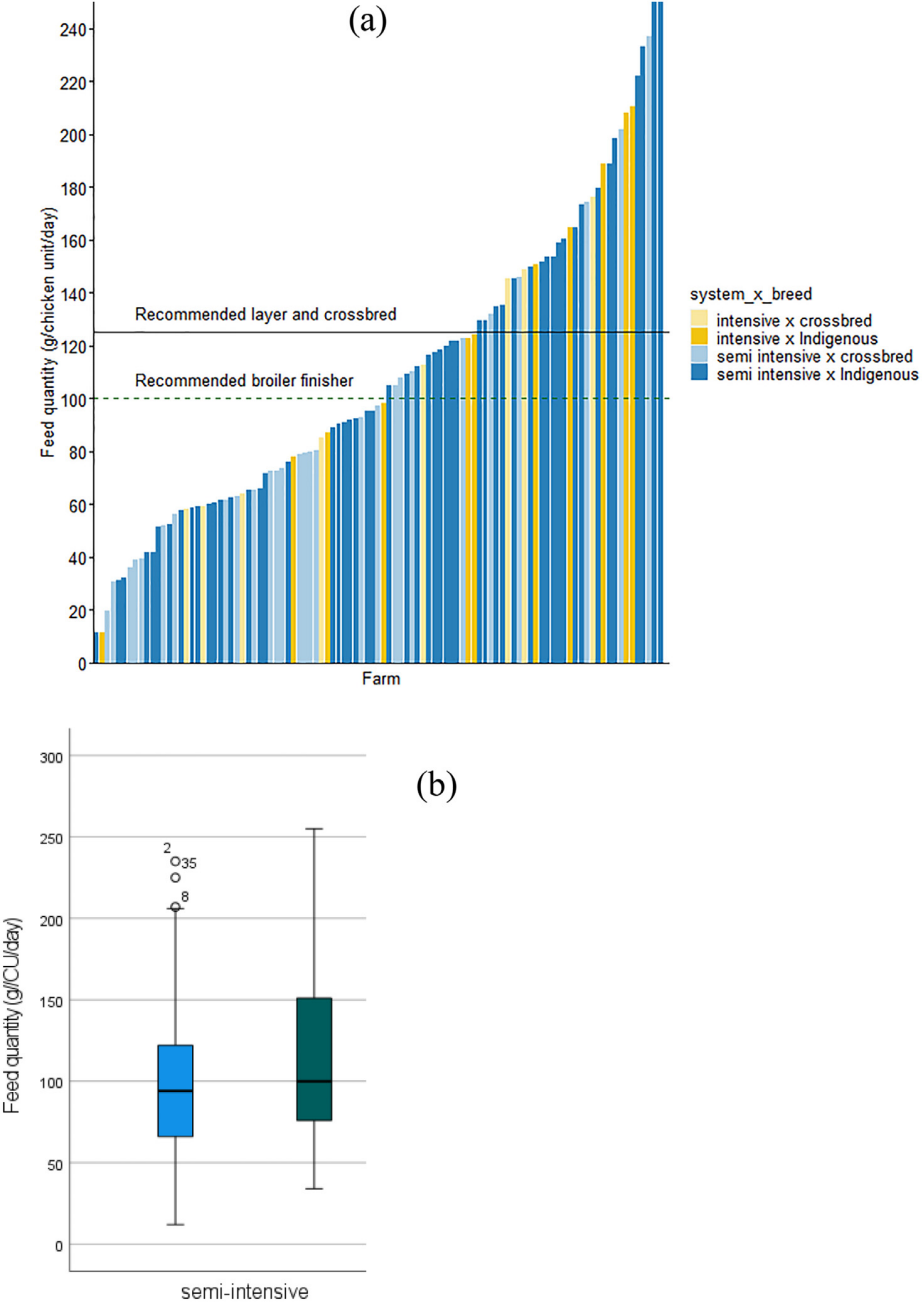
Comparison of feed quantity recommended for broiler, layers, and improved crossbred (A) and the quantity of feed fed to indigenous and improved crossbred chicken units per day under semi-intensive and intensive systems (B) in Iringa municipality.

**Figure 5:**
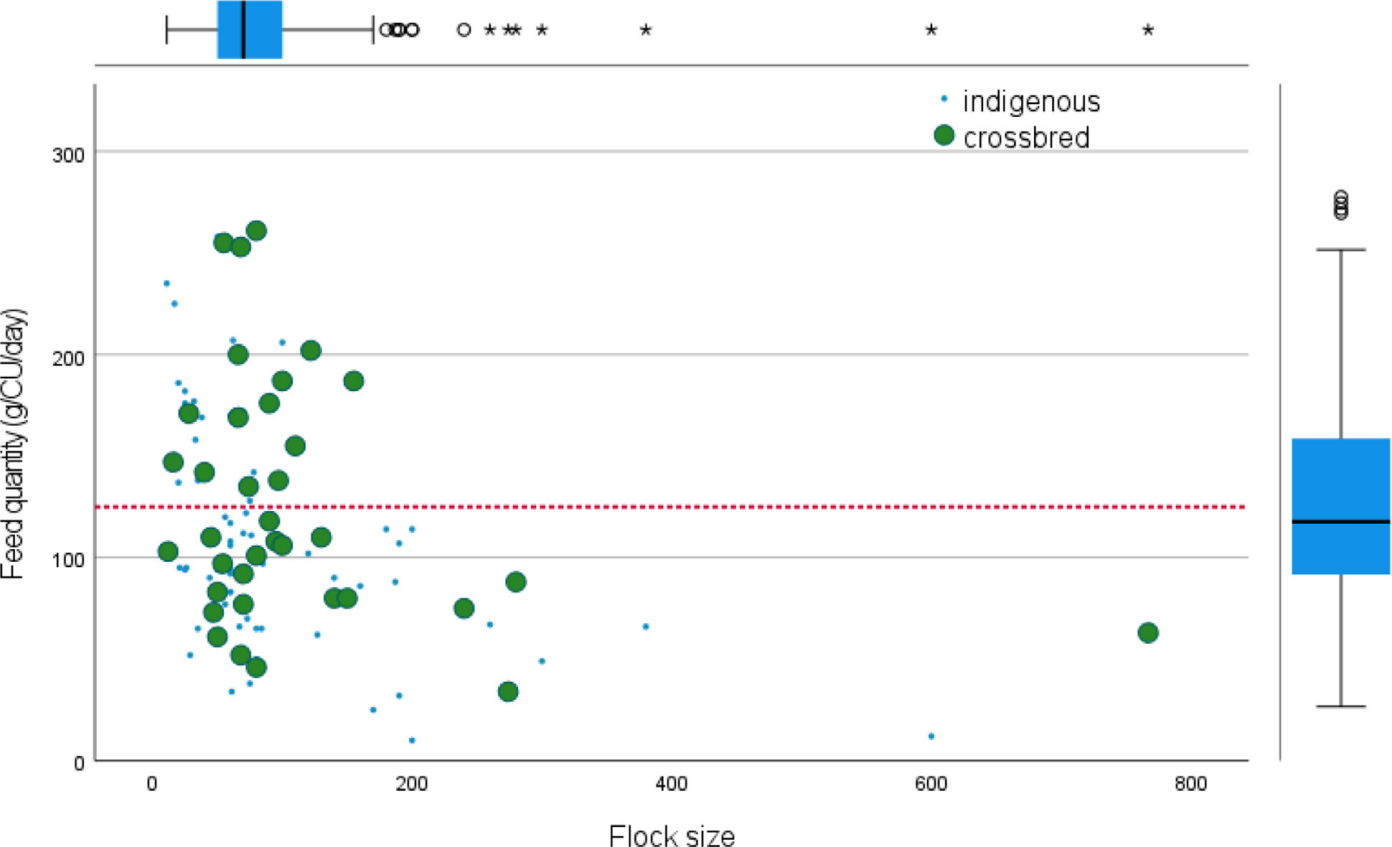
Relationship between feed quantity and flock size (chicken unit). The optimum recommended feed quantity for a matured chicken is provided in a red dotted line (≥125 g/d). The box plots indicate the mean flock size and feed quantity, respectively.

When considering the concept of CU, 40% of the interviewed households met the optimum recommendations of ≥125 g of feed required for a CU (mature laying hen) per day (Figures 4 and 5). Within the intensive system, 50% of farmers supplied above the average of the recommended feed ration for mature laying hens. When considering the quantity of feed required for broiler finisher chickens, 50% of the households met the requirements as recommended by the breeding company (100 g/CU per d). All farms with ≥200 chickens fed their chickens less than the recommended quantity of feed (125 g/CU per d) for layers and crossbred chickens (Figure 5).

### Feed Nutritional Quality

**Figure 6:**
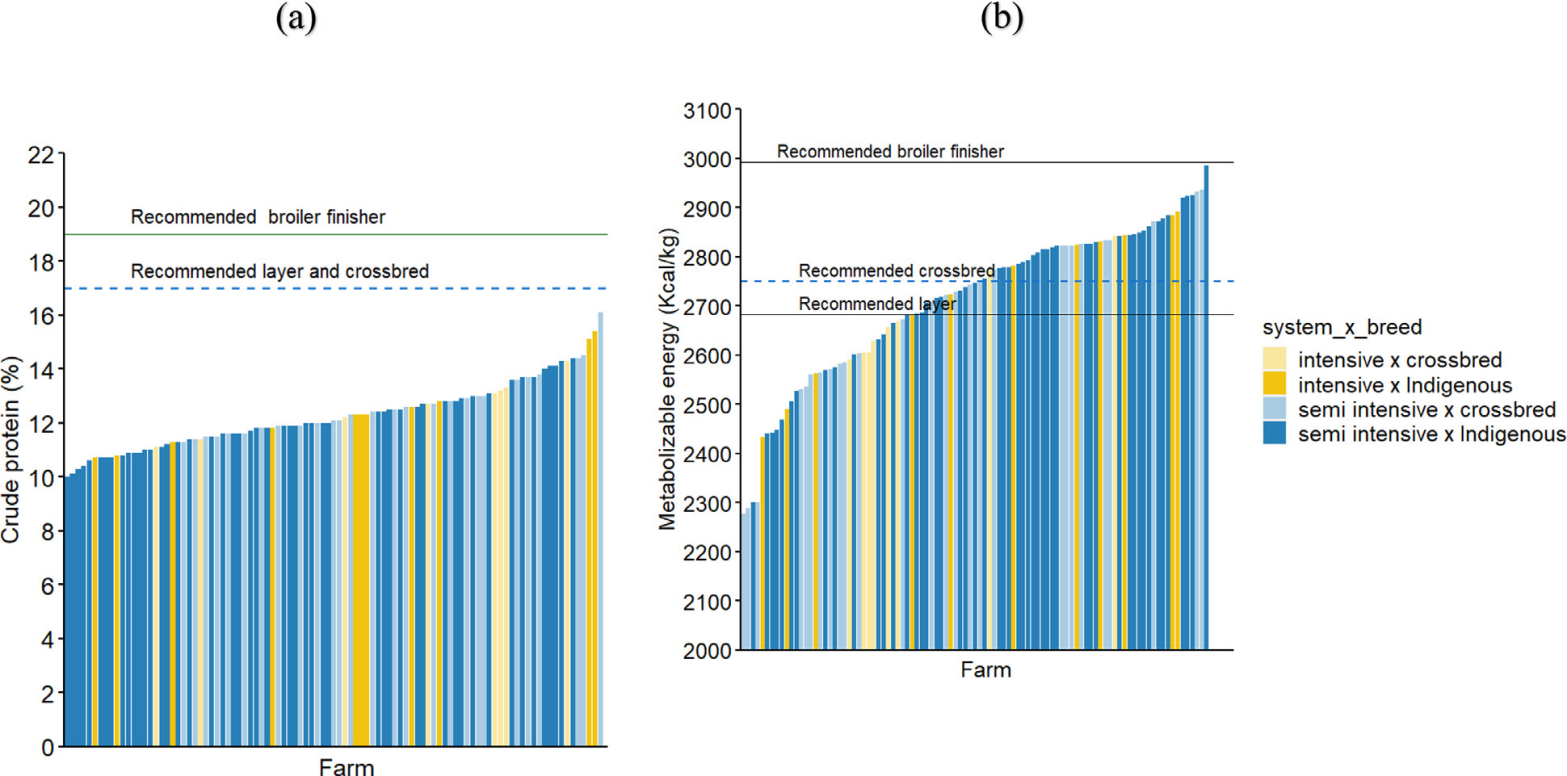
Nutritional quality of the feed samples from the study area in terms of crude protein (a) and metabolizable energy (b) based on the laboratory analysis and the comparison with the average recommended values from the East African Standards, feed calculator App and international breeding companies.

Overall, none of the feed samples analyzed met the recommended nutritional standards for crude protein for broilers, layers and improved crossbred chickens while 60 and 47% of the samples had the recommended metabolizable energy content for layers and improved crossbred chickens (Figure 6). When considering the recommendations for broiler finisher feed, none of the feed samples had the recommended metabolizable energy and crude protein content. For the essential amino acids, 48 and 10% of the samples had the recommended lysine and methionine or cysteine concentrations for layers and improved crossbred chickens, respectively.

**Figure 7:**
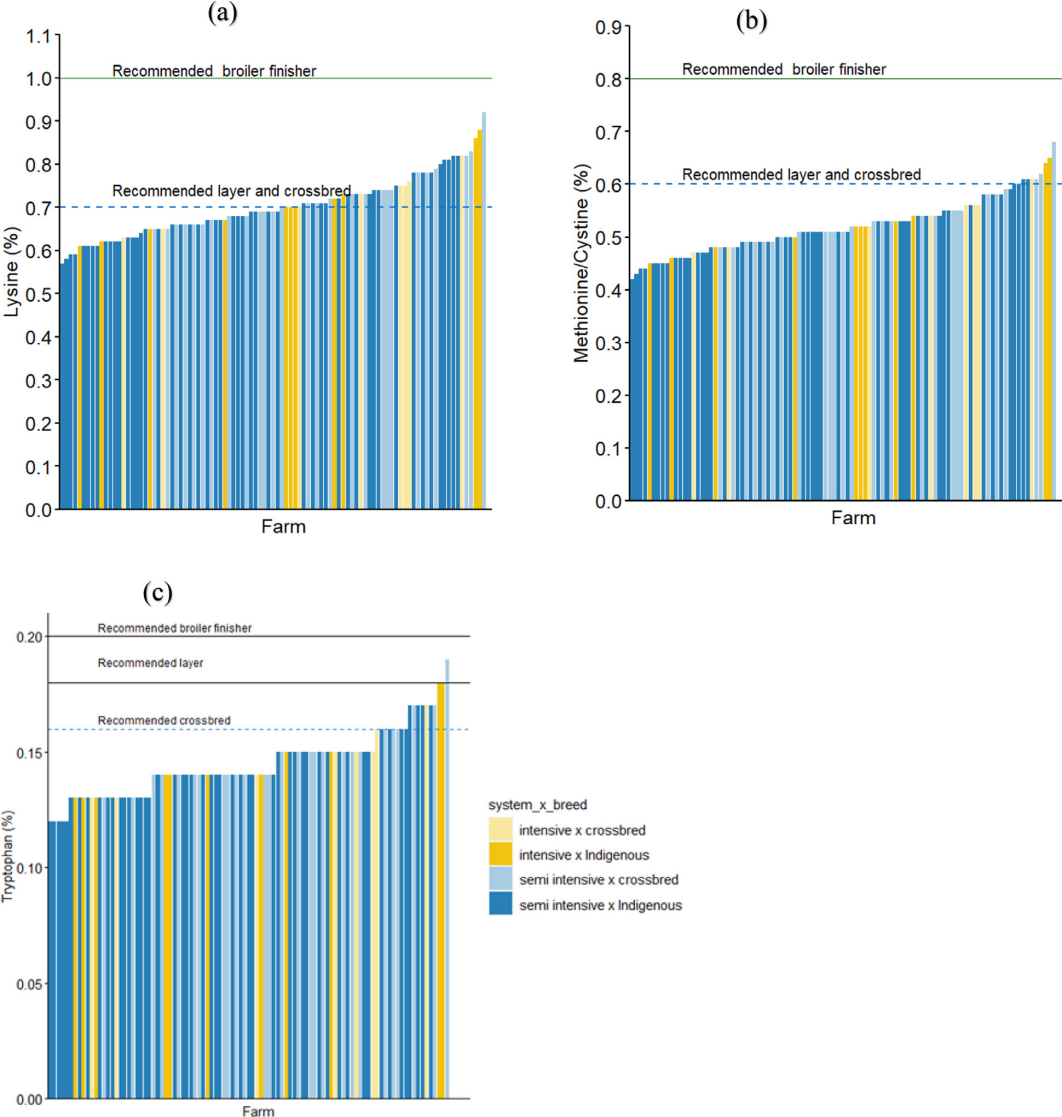
Essential amino acids concentration of the feed samples, that is, lysine (a), methionine/cysteine (b), and tryptophan (c). The horizontal lines indicate the recommendations from the Tanzania/East African Standards, feed calculator App and international breeding companies.

When considering the recommendations for broiler finisher feed, none of the feed samples reached the recommended concentration of the essential amino acids (Figure 7). When considering the recommendations for layers and improved crossbred feed, 3 and 19% of the feed samples reached the minimum tryptophan concentration, respectively (Figure 7 and Table 2).

### Aflatoxin and Moisture Content

**Figure 8:**
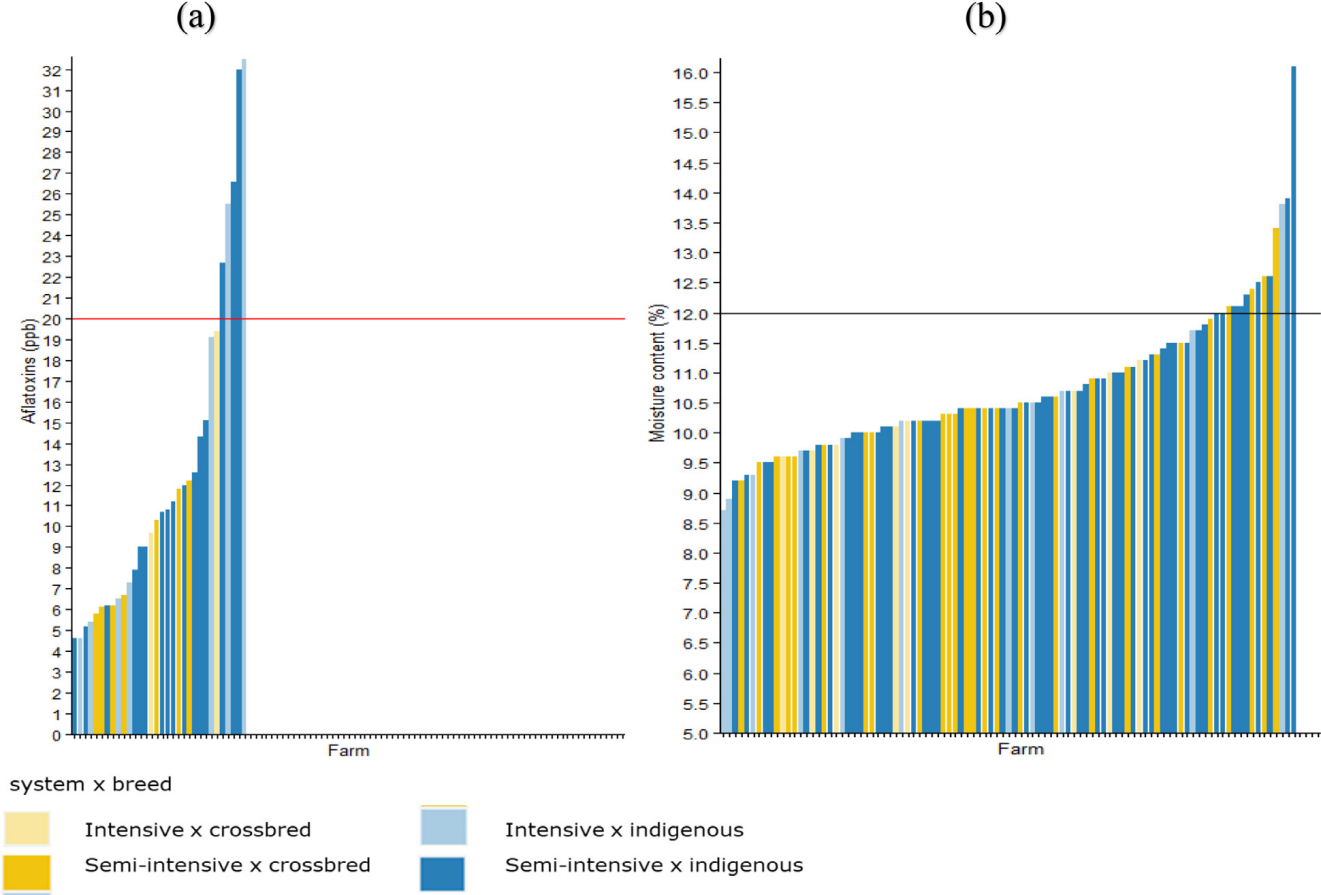
(A) Aflatoxin and (B) moisture content assessment in relation to the maximum limits (horizontal lines) set by the Tanzania/East African Standards, respectively.

Of all 101 respondents interviewed, 1% was aware of aflatoxin and its effects on animal and human health. All feed samples analyzed contained a detectable concentration of aflatoxins of which 16% exceeded the East Africa Community standards of 20 µg/kg. The highest concentrations of aflatoxins were observed in feed samples collected from farms raising indigenous chickens in both semi-intensive and intensive systems, with 32.5 µg/kg and 26.6 µg/kg, respectively (Figure 8). Twelve percent of the collected samples had excess moisture content in the semi-intensive and intensive systems.

## DISCUSSION

The intensification of the poultry industry in Tanzania involves the transition from keeping small numbers of free-range indigenous chickens per farm to large flocks of multiple breeds or improved breeds of chickens under semi-intensive and intensive systems (Wilson et al., 2022). The newly introduced crossbred, that is, Kuroiler and Sasso breeds have shown potential in the highlands and lowlands of Tanzania (Sanka et al., 2020; Guni et al., 2021). The present study explored a major component of the yield gaps in the poultry industry in Tanzania by first analyzing the effects of rearing system and breed of chickens and interaction effects on production parameters (body weight and egg production). In the next step, we analyzed the effects of the rearing systems and chicken breed and their interaction effect on feed quantity and quality. To the best of our knowledge, there are no relevant simulation models for estimating the potential production of chickens which would be needed to estimate the yield gap (Van de Ven et al., 2003; van Ittersum et al., 2013; van der Linden et al., 2021). Considering the crucial importance of feed in closing the yield gap in chicken production, we focused on the yield that could be attained by closing feed gaps (both quantity and quality).

Most chicken farmers keeping dual-purpose chickens in Tanzania prefer locally made feeds or feed ingredients to reduce the costs of production and evade expensive commercial feeds, which are mostly not available in small quantities (Wilson et al., 2022). Our results show that the feeds are indeed of low quality for all breeds but provided in inadequate quantity, especially for indigenous chickens, suggesting differentiated improvements to feeding practices are required to reach the potential production of dual-purpose chickens of both breeds, and hence reduce the yield gap. Several studies reported limited access to high-quality feed and feed ingredients as the major constraint limiting chicken production and productivity in Tanzania (Longo Joseph, 2019; Naggujja et al., 2020; Enahoro et al., 2021), but they did not describe local feeding strategies in different production systems in detail. In comparison with the East African standards and international breeding companies’ recommendations, we found that most dual-purpose chickens in the study area received feeds particularly low in crude protein and essential amino acids. Research shows that a well-managed flock of improved dual-purpose crossbred chickens may reach 1,800 g body weight and egg production potential of about 95% (weekly lay/flock, laying eggs of ≥52 g) at peak lay (Hendrix Genetics, 2022). Nevertheless, when considering the realistic potential weekly lay percentage (80%) that can be attained by most farmers (van der Linden et al., 2015), our findings show that only 50% of the interviewed farms attained the potential body weight of mature chickens and 40% of the farms met the potential weekly lay %, in major part due to the poor quantity and quality of feed provided.

Our findings show that the poor quality of the feed provided is partly due to the prices of feed ingredients, as the most important ingredients, that is, the protein sources, essential amino acids, and premixes are expensive. We found that maize bran, sunflower seedcake and fishmeal are the main sources of energy and protein in the study area. Soybean cake is also an important source of protein produced within the region, but its utilization in chicken feed formulation is limited due to a lack of efficient processing facilities (Wilson et al., 2021). As a result, the locally produced soybean grain is exported to the neighboring countries for processing and soybean meal is imported at prices that are not affordable for most chicken farmers.

Apart from feed quantity and quality, attaining the potential production requires proper animal breeding and a favorable environment, that is, proper housing (clean, well-ventilated, and with regulated temperature) (van der Linden et al., 2015). There is also a crucial need to consider the acceptable levels of animal welfare and growth-reducing factors such as water quality, housing, disease and parasite prevalence and feed contamination (aflatoxin). Only feed samples collected from the farms raising indigenous chickens exceeded the maximum aflatoxin limits (>20 µg/kg) in both systems, posing high risks to chicken productivity and human health. Despite low moisture content observed in feed samples from most farms, there are dangers of aflatoxin contamination due to limited awareness of farmers on the presence, effects and control of aflatoxins. The most common feed ingredients used in formulating chicken feed include maize and sunflower seed cake, which are among the feeds that are prone to aflatoxin contamination (Mwakosya et al., 2022). Research shows clear evidence of the effects of aflatoxins on depressed laying performance, feed intake, eggshell thickness, and egg hatchability, along with the deposition of aflatoxin residues in eggs and meat (Jia et al., 2016; Al-Ruwaili et al., 2018; Coppock et al., 2018). We did not observe a significant relationship between aflatoxin content and egg production (weekly lay %) in the present study, perhaps due to the small sample size. Free range chickens are more exposed to aflatoxin contamination than semifree range chickens since they rely on scavenging with exposure to contaminated feeds (Tarus, 2019). Therefore, since most consumers in Tanzania prefer products from indigenous free-range chickens (Sanka and Mbaga, 2014; Naggujja et al., 2020), there is a need for interventions in chicken management and feed quality management to reduce aflatoxin residues in chicken meat and eggs.

Alternative protein sources including insects and insect larvae have been proposed as replacements for fish meal in tropical countries, particularly in monogastric feeds (Vernooij et al., 2019; Oosting et al., 2022). Recently, some companies have invested in processing city waste into valuable proteins through black soldier fly (**BSF**) production in major towns and cities (Vernooij et al., 2019; Isibika, 2022; Limbu et al., 2022), but the scale at which it could contribute to the growing-chicken industry in Tanzania is unclear. The advent of open-access feed-calculator mobile applications (e.g., FeedCalculator, 2022) provides an opportunity for small-scale chicken producers to design better feeds for their chickens. Further improvements of the tool should focus on the inclusion of alternative protein sources and locally produced feed ingredients.

## CONCLUSIONS

In line with the Tanzania Livestock Master Plan, our findings reveal a large feed gap in current chicken production. Despite the recent intensification of the chicken industry in Tanzania through the introduction of improved and exotic breeds, there is a high yield gap in chicken production. Our results highlight the importance of closing feed gaps (both quantity and quality) to meet the increasing demand for chicken meat and eggs.
